# Web-Based Psychological Interventions for People Living With and Beyond Cancer: Meta-Review of What Works and What Does Not for Maximizing Recruitment, Engagement, and Efficacy

**DOI:** 10.2196/36255

**Published:** 2022-07-08

**Authors:** Monica Leslie, Lisa Beatty, Lee Hulbert-Williams, Rosina Pendrous, Tim Cartwright, Richard Jackson, The Finding My Way UK Trial Steering Group, Nicholas J Hulbert-Williams

**Affiliations:** 1 Department of Psychology Edge Hill University Ormskirk United Kingdom; 2 College of Education, Psychology & Social Work Flinders University Adelaide Australia; 3 Liverpool Clinical Trials Centre University of Liverpool Liverpool United Kingdom; 4 see Acknowledgments; 5 Centre for Contextual Behavioural Science School of Psychology University of Chester Chester United Kingdom

**Keywords:** cancer, neoplasms, survivors, psychosocial oncology, internet-based intervention, psychosocial intervention

## Abstract

**Background:**

Despite high levels of psychological distress experienced by many patients with cancer, previous research has identified several barriers to accessing traditional face-to-face psychological support. Web-based psychosocial interventions have emerged as a promising alternative.

**Objective:**

This meta-review aimed to synthesize evidence on recruitment challenges and enablers, factors that promote engagement and adherence to web-based intervention content, and factors that promote the efficacy of web-based psychosocial interventions for patients with cancer and cancer survivors.

**Methods:**

We conducted a systematic search of previous reviews that investigated the recruitment, engagement, and efficacy of web-based and app-based psychosocial interventions in adult patients with cancer and cancer survivors. We searched PubMed, CINAHL, PsycINFO, and the Cochrane Library database for relevant literature. The search terms focused on a combination of topics pertaining to neoplasms and telemedicine. Two independent authors conducted abstract screening, full text screening, and data extraction for each identified article.

**Results:**

A total of 20 articles met eligibility criteria. There was inconsistency in the reporting of uptake and engagement data; however, anxiety about technology and perceived time burden were identified as 2 key barriers. Web-based psychosocial oncology interventions demonstrated efficacy in reducing depression and stress but reported weak to mixed findings for distress, anxiety, quality of life, and well-being. Although no factors consistently moderated intervention efficacy, preliminary evidence indicated that multicomponent interventions and greater communication with a health care professional were preferred by participants and were associated with superior effects.

**Conclusions:**

Several consistently cited barriers to intervention uptake and recruitment have emerged, which we recommend future intervention studies address. Preliminary evidence also supports the superior efficacy of multicomponent interventions and interventions that facilitate communication with a health care professional. However, a greater number of appropriately powered clinical trials, including randomized trials with head-to-head comparisons, are needed to enable more confident conclusions regarding which web-based psychosocial oncology interventions work best and for whom.

**Trial Registration:**

PROSPERO CRD42020202633; https://www.crd.york.ac.uk/prospero/display_record.php?RecordID=202633

## Introduction

### Background

The physical, financial, and existential challenges of living with cancer pose significant threats to psychological well-being [1-3]. Up to 52% of patients with cancer report clinically significant psychological distress [2,4], which can affect quality of life (QoL) [5] and is associated with higher mortality in some cancers, even after controlling for age, sex, education, socioeconomic status, BMI, smoking, and alcohol intake [6].

A range of therapeutic approaches are commonly used to support the psychological well-being of patients with cancer and cancer survivors [7,8]. For example, cognitive behavioral therapy (CBT) is currently considered the gold standard treatment for managing distress and improving psychological outcomes in populations living with or beyond cancer [7]. CBT techniques, as applied to patients with cancer and cancer survivors, commonly include psychoeducation, thought monitoring and challenging exercises, and activity planning and pacing activities [8,9]. Other interventions include mindfulness-based interventions and acceptance and commitment therapy [7], as well as meaning-centered psychotherapy, which has particularly promising evidence for patients with advanced cancer [8]. In addition, a growing number of guided self-management interventions aim to support psychological well-being based on the principles of self-determination theory [10,11]. These interventions aim to support the basic needs of patients for autonomy, competence, and relatedness [12].

However, despite the range of available therapeutic options, many patients experience difficulties in accessing traditional face-to-face psychological support [13]. For example, a previous systematic review identified that 17% of patients experienced transport and parking as barriers to psychosocial care [13]. In addition, a recent scoping review further highlighted that distance to the treatment center can increase the likelihood of disengagement with psychosocial support [14]. To overcome these barriers, researchers have increasingly investigated web-based psychosocial interventions for patients with cancer [15], including self-guided app-based programs, self-guided web-based programs, web-based peer support, videoconferencing with a health care professional, and combinations of these approaches [16,17].

Qualitative studies have indicated that cancer survivors have positive attitudes toward internet-based interventions [18,19], and several recent reviews of web-based psychosocial interventions for cancer survivors have been published [20-22]. However, these reviews differ in scope, considering varying populations and differing types of web-based and app-based psychosocial interventions. Consequently, a wide range of conclusions have been reached across existing systematic reviews, varying from negligible effects for QoL [16] to significant effects reported for all studies in which depressive symptoms were described as an outcome [22]. This wide discrepancy establishes a conflicting evidence base.

In addition, there is a dearth of evidence on the factors promoting uptake and engagement with web-based psychosocial interventions for people living with and beyond cancer [23]. Previous evidence examining the uptake of psychological interventions for patients with cancer has found that telephone-based interventions are more popular than face-to-face delivery [24], suggesting that removing barriers related to transport and commuting time may be important [13]. However, it is unclear which features of web-based psychosocial interventions are likely to enhance the perceived utility and acceptability of some interventions. Given the previous evidence of dose-response effects, such that greater module completion is associated with more improved outcomes in web-based interventions [25], it is also important to identify factors that promote greater engagement.

Meta-reviews offer an effective approach for addressing the discrepancies in the existing review findings [26]. In particular, where several recent reviews have been conducted on a single or highly similar topic, meta-reviews allow the integration of data and emergence of consensus to better inform clinical practice and research design decisions [27,28].

### Study Aim and Objectives

This meta-review aimed to identify and critically appraise existing systematic reviews of web-based and app-based psychosocial interventions for patients with cancer and cancer survivors. Our specific objectives were to synthesize evidence on the following: (1) recruitment challenges and enablers; (2) factors that promote engagement and adherence; and (3) factors that promote efficacy in improving psychosocial outcomes, including distress, depressive symptoms, anxiety, stress, QoL, and subjective well-being.

## Methods

### Study Method

We conducted a systematic search to identify existing reviews of web-based and app-based psychosocial interventions for cancer survivors, according to recommended methodological guidance [27]. This systematic meta-review was preregistered on PROSPERO (CRD42020202633).

### Search Strategy

We searched PubMed, CINAHL, Cochrane Review Library, and PsycINFO databases. These databases are respectively associated with different search functionalities, such that PubMed and the Cochrane Review Library provide the option to search Medical Subject Headings terms to organize and retrieve records using a common hierarchically organized vocabulary. CINAHL uses a similar but separate taxonomy of content organized under CINAHL Subject Headings. By contrast, PsycINFO does not have functionality for searching records using a standardized vocabulary system. Therefore, we optimized our search terms for each database to best use the functionality offered by each database. Search terms and Medical Subject Headings focused on a combination of neoplasms (and related terms), reviews (and related terms), and internet-based interventions or telemedicine. Multimedia Appendix 1 provides the full search strategy.

We conducted a systematic search on August 5, 2020. We did not specify time limits in our systematic search given that the earliest possible publication of relevant publications was naturally limited by the relatively recent emergence of web-based psychosocial interventions.

Following the identification of eligible articles in our database searches, we screened the reference lists of these articles to identify other eligible articles.

### Inclusion Criteria

Articles were eligible for inclusion if they met the Population, Intervention, Comparison, Outcomes, and Study design criteria [29] shown in Textbox 1 and were published in English.

**Inclusion criteria.**

**Inclusion criteria**

- *Population*: adults, defined as participants meeting the minimum age of independent research consent in their respective country (ie, aged 16 or 18 years), who received a diagnosis of cancer at some point in their lives.
- *Interventions*: internet and mobile app-based psychosocial interventions with a primary aim of improving psychological outcomes through the provision of interactive psychological or social support. Information-only or noninteractive psychoeducational resources were excluded.
- *Control group*: studies with any type of control group or single-arm trials without a control group.
- *Outcomes*: psychosocial outcomes including distress, depressive symptoms, anxiety, stress, QoL, and subjective well-being.
- *Study design*: systematic reviews with narrative syntheses or meta-analyses.
- Published in English

### Exclusion Criteria

Articles were excluded if they met any of the criteria shown in Textbox 2.

**Exclusion criteria.**

**Exclusion criteria**

- Included data from populations with a current or previous diagnosis of cancer and other clinical groups, where data from cancer populations alone could not be extracted.
- Included data from children and adults, where the data from adult populations alone could not be extracted.
- Included data from studies that combined face-to-face with web-based interventions, where the data from the web-based intervention alone could not be extracted.
- Included interventions that were information-only or exercise-based interventions.
- Were a nonpeer-reviewed publication or book chapter.

### Article Screening

After deduplication, title and abstract screening was performed to confirm article eligibility, recording reasons for article exclusion where applicable. Each abstract was independently checked by 2 members of the team.

This process was repeated for articles that had undergone full-text screening. In all, 2 authors (ML and TC) screened the reference lists of eligible articles to identify any additional articles that may not have been identified in the primary systematic search process. We deemed systematic reviews to have an insufficient focus on the Population, Intervention, Comparison, Outcomes, and Study design criteria, where the specificity of the inclusion criteria resulted in the inclusion of only one relevant original study. For example, a systematic review that included only one relevant original study owing to an exclusive focus on CBT-based interventions would be excluded [30]. The exception to this rule was where systematic reviews focused on a specified intervention format (ie, internet-based self-help interventions) [31], given our interest in comparing different modalities of web-based interventions.

### Data Extraction

We first piloted our data extraction process to confirm consistency across reviewers. A standardized data extraction form was used to aid independent data extraction (Multimedia Appendix 1). Data extraction for each included paper was conducted in duplicate by 2 members of the review team. Any discrepancies in data extraction were resolved by a third author.

### Quality Assessment

The quality of each included review was assessed independently by 2 authors against the 27 PRISMA (Preferred Reporting Items for Systematic Reviews and Meta-Analyses) statement criteria [32], as shown in Multimedia Appendix 2 [16,17,21,31,33-48]. The PRISMA statement criteria include the assessment of the risk of bias within and across studies. We decided a priori to include all eligible reviews, including those meeting relatively fewer PRISMA criteria, given that these reviews might feasibly contribute to the divergence of findings reported in the literature thus far and were thus relevant to account for in this meta-review. However, although poor-quality reviews were not excluded, our synthesis accounted for relevance and quality in our discussion of similarities and differences reported. We only included articles that performed a systematic search for relevant original studies to minimize the likelihood of selection bias in our data set [49].

### Analysis

Narrative synthesis of the results of the included reviews was conducted. This considered the quality of both (1) the systematic review and (2) the original studies included within those reviews. Evidence was synthesized regarding the range of interventions tested; the overall uptake of interventions and factors that promote intervention uptake or trial recruitment; the overall adherence to, and engagement with, interventions reviewed (including facilitating factors for intervention adherence); overall efficacy (including facilitating factors for intervention efficacy); and information on the suitability of outcome assessments. Given that most eligible systematic reviews reported a narrative synthesis of trial outcomes, most of the data included in this study were qualitative in nature. Thus, we opted for an inductive thematic analysis of the review findings, which has been identified as a rigorous method of synthesizing qualitative data while remaining faithful to the original data [50].

## Results

### Systematic Search Results

**Figure 1:**
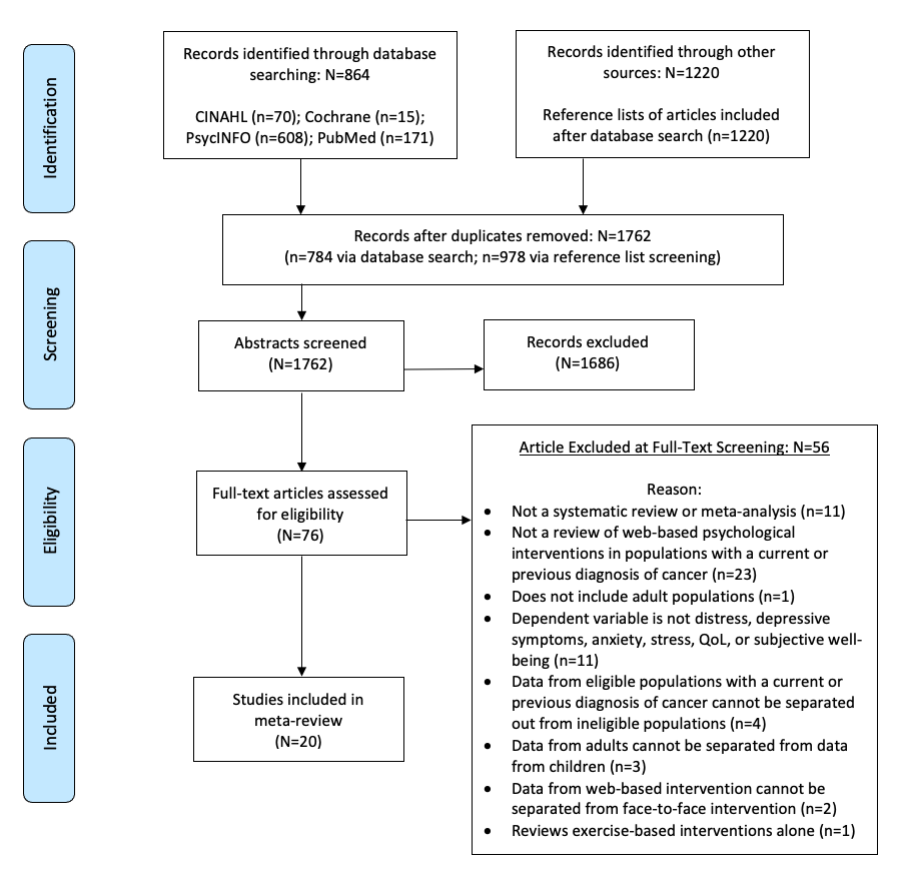
PRISMA (Preferred Reporting Items for Systematic Reviews and Meta-Analyses) diagram.

Figure 1 summarizes the screening and eligibility process. The initial search yielded a total of 864 articles. After deduplication (80 articles), 784/864 articles (90.7%) underwent abstract screening. Agreement between reviewers for inclusion and exclusion decisions at the abstract screening stage was 92%, with discrepancies resolved by a third author. A total of 74 articles underwent full-text screening, with a 78% agreement rate between reviewers. Discrepancies at this stage were discussed at an audit meeting of 4 authors (ML, NJHW, LHW, and RP), with final inclusion decisions reached by consensus. A total of 19 reviews were selected for inclusion based on this initial search (Figure 1 provides the reasons for exclusion).

Reference lists of the 19 included articles were examined (N=1220 papers). After removing 242 (19.84%) duplicates, titles of the remaining 978 (80.16%) papers were screened for eligibility. An additional paper identified from the reference lists met the inclusion criteria for this review [33]. Therefore, this meta-review included 20 articles: 5 (25%) meta-analyses, 14 (70%) systematic reviews with narrative synthesis, and 1 (5%) integrative review including both quantitative and qualitative studies. Of the 20 included reviews, 5 (25%) exclusively reviewed randomized controlled trials (RCTs), 4 (20%) reviewed RCTs and quasi-experimental studies, 2 (10%) reviewed RCTs and single-arm feasibility studies, 4 (20%) reviewed both quantitative and qualitative studies, and 5 (25%) did not specify the study design in their inclusion criteria. The year of publication of the included reviews ranged from 2009 to 2020.

Most of the original studies were included in only one review. The full list of original studies included in the systematic reviews, including the frequency with which each study was included in multiple reviews, is presented in Multimedia Appendix 3 [16,17,21,31,33-48,51-57].

### Review Quality

PRISMA items 13, 14, 16, 20, 21, and 23 were deemed not relevant for systematic reviews with narrative syntheses. Therefore, for each paper, we calculated the percentage of applicable PRISMA criteria met, which ranged from 52.4% to 100% (average 77.9%, SD 13.7%, representing moderate review quality) [58]. A total of 6 criteria were met in all 20 papers. Only 4 systematic reviews met criterion 5 (“Indicate if a review protocol exists, if and where it can be accessed [eg, web address], and, if available, provide registration information including registration number”) and 7 met criterion 22 (“Present results of any assessment of risk of bias across studies”). Further details are provided in Table 1.

**Table 1:** Included reviews.

Study	Number of applicable PRISMA^a^ criteria met, n/N (%)	Type of review	Population	Intervention	Comparison	Relevant outcomes captured	Study designs included	Outcome of internet-based psychosocial oncology interventions
Agboola et al [34]	15/21 (71)	Systematic review with narrative synthesis	Patients with cancer	Telephone-based interventions (N/A^b^), telephone-based interventions in conjunction with web-based systems, and web-based interventions	Any	Depression and QoL^c^	RCT^d^	Among internet-based interventions, 4/4 (100%) found improvements in depression and 2/2 (100%) found improvements in health-related QoL.
Beatty and Lambert [31]	16/21 (76)	Systematic review with narrative synthesis	Adults (aged ≥18 years) with a chronic physical health condition	Self-help internet-based psychosocial therapeutic interventions; within cancer: only iCBT^e^	Any	Distress, QoL, and well-being	RCT, quasi-randomized trial, or feasibility RCT study	Did not find significant improvements in distress, QoL, or well-being.
Chen et al [35]	23/27 (85)	Meta-analysis	Patients with breast cancer	Telehealth intervention, defined as that delivered by telephone (N/A), internet-based interfaces, or other remote information systems, which can overcome the barriers of time and distance (N/A)	Usual care alone	QoL, depression, anxiety, distress, and perceived stress	RCT	Meta-analysis found a significant between-group effect for depression but not for QoL.
Forbes et al [36]	21/21 (100)	Systematic review with narrative synthesis	Prostate cancer survivors	Web-based interventions designed to improve supportive care outcomes	Included single-arm studies and studies with any comparison group	Cancer-related distress, health-related QoL, and depressive symptoms	Single-arm feasibility or acceptability study or randomized trial	Mixed findings for significant between-group effects on distress (1/2, 50% studies). Significant pre-post effects for both depression and QoL but no significant between-group effects for depression and mixed effects for QoL.
Fridriksdottir et al [21]	13/21 (62)	Systematic review with narrative synthesis	Adult patients with cancer	Internet or web-based interventions	Any type of control group (standard care or wait-list or usual face-to-face care or different types of internet-based intervention)	Distress, anxiety, and depression	RCT, pilot RCT, or quasi-experimental studies	Mixed evidence for between-group effects anxiety. Few studies finding significant intervention effects for distress (2/6, 33% studies) and depression (2/7, 28% studies).
Goliță and Băban [37]	19/21 (90)	Systematic review with narrative synthesis	Adults (aged >18 years) diagnosed with cancer in curative treatment or survivorship phase	Web-based psychotherapeutic interventions	Wait-list, placebo, usual-care, treatment-as-usual, or standard-of-care conditions	Psychological distress and QoL	RCT	In all, 8/11 (73%) studies found significant between-group effects for distress, and 4/10 (40%) studies found significant between-group effects for QoL.
Griffiths et al [17]	13/21 (62)	Systematic review with narrative synthesis	Individuals part of internet support groups (studies relevant to cancer separated out in results)	Online support groups with a discussion focus on health or psychology	Any or none	Depressive symptoms	Quantitative or qualitative studies	In all, 4/4 (100%) studies without a control group found significant pre-post effects for depression, and 1/3 (33%) with a control group found significant between-group effects.
Hong et al [38]	15/21 (71)	Systematic review with narrative synthesis	Adult cancer survivors	Web-based support or resources	Any or none	Distress, QoL, stress, depression, health-related QoL, psychological well-being, and emotional well-being	Quantitative or qualitative studies	No positive outcomes found for distress, QoL, or well-being compared with control.
Ihrig et al [39]	17/21 (81)	Systematic review with narrative synthesis	Men diagnosed with prostate cancer and their caregivers and significant others	Web-based peer-to-peer support within online support groups or other forms of interactive peer-to-peer communication in social media	Any (eg, face-to-face support groups, psychosocial interventions, standard care, other, or none)	QoL	Not specified	In all, 1/1 (100%) study investigating QoL found significant between-group effects at 6 weeks but not 8 weeks.
Larson et al [40]	20/27 (74)	Systematic review and meta-analysis	Adult patients with cancer, in active treatment	Telehealth or telemedicine, including, but not limited to, telephone calls (N/A) and web-based interventions; focused on emotional support or self-management of symptoms through counseling, educational intervention, or telepsychiatry	Any or none	QoL	Not specified	In all, 1/3 (33%) studies found significant pre-post effects for QoL.
McAlpine et al [41]	15/21 (71)	Systematic review with narrative synthesis	Adult cancer survivors	Web-based interactive intervention for patient education, to connect patients with each other or connect patients with their health care clinicians	Any or none	QoL, distress, and stress	Not specified	In all, 1/1 (100%) study found favorable results for stress, 1/2 (50%) found favorable results for anxiety, and 3/6 (50%) studies found favorable results for depression. In addition, 0/2 (0%) studies found favorable results for distress, and 2/5 (40%) studies found favorable results for QoL.
Moradian et al [42]	13/21 (62)	Systematic review with narrative synthesis	Adult patients with cancer (aged >18 years) receiving chemotherapy	eHealth, web, and app-based interventions	Any	Health-related QoL, distress, anxiety, and depression	Randomized or nonrandomized controlled trials and pre-post or quasi-experimental intervention studies with a comparison group	Only studies reviewing distress and depression found significant between-group effects. In all, 0/1 (0%) studies found significant between-group effects for QoL, and 1/2 (50%) study found significant between-group effects for anxiety.
Paul et al [43]	11/21 (52)	Systematic review with narrative synthesis	Patients with common chronic conditions	Web-based interventions designed to improve psychological well-being or QoL	Any or none	Depression, anxiety, QoL, psychological well-being, emotional well-being, and social well-being	Not specified	In all, 2/2 (100%) studies found significant pre-post improvements in depression, stress, and QoL, and 0/2 (0%) studies found positive effects for well-being.
Qan’ir and Song [44]	18/21 (86)	Systematic review with narrative synthesis	Patients with prostate cancer	Technology-based interventions	Any	Depression, anxiety, and QoL	RCT or quasi-experimental research design	In all, 1/4 (25%) study found significant improvements for anxiety, 2/6 (33%) studies found significant improvements for depression, and 1/6 (17%) study found significant improvements for health-related QoL. A study found significant between-group improvement for depression.
Seiler et al [45]	25/27 (93)	Systematic review with meta-analysis	Cancer survivors	eHealth or mHealth^f^ interventions	Any or none	Health-related QoL, depression, and psychological distress	RCT, cross‐sectional survey, prospective case‐control or cohort study, pilot study, longitudinal observational study, or qualitative survey	Meta-analysis found a significant between-group effect for depression and health-related QoL. In all, 1/2 (50%) study found a significant decrease in distress.
Toivonen et al [33]	19/21 (90)	Systematic review with narrative synthesis	Individuals with chronic physical health conditions	Web-based mindfulness-based interventions	Any or none	Stress and psychological distress	RCTs, non-RCTs, and uncontrolled studies	A study found significant pre-post effects for psychological distress. Another study found significant between-group effects for stress.
Triberti et al [46]	15/21 (71)	Systematic review with narrative synthesis	Patients with breast cancer	Internet-based interventions, support groups, and apps	Any or none	QoL, depression, stress, anxiety, and emotional well-being	Not specified	Overall positive effects found for depression, anxiety, stress, QoL, and emotional well-being.
Wang et al [47]	25/26 (96)^g^	Systematic review and meta-analysis	Patients with cancer	Internet-based psychoeducation interventions	Standard or usual care or a conditional control group	QoL, depression, and distress	RCT or clinical controlled trial	Meta-analysis found significant between-group effects for depression but not distress or QoL.
Xu et al [16]	26/27 (96)	Systematic review and meta-analysis	Adult patients with cancer	eHealth-based health care	Non–eHealth-based control conditions	QoL	RCT	Meta-analysis did not find significant between-group effects for QoL.
Zhu et al [48]	14/21 (67)	Integrative review	Women with breast cancer	Internet and app-based support and symptom management programs	Any or none	QoL and depression	Quantitative or qualitative studies	In all, 1/2 (50%) study found significant pre-post effects for QoL. A study found that QoL improved more in the control group. A study found a significant between-group effect for depression.

^a^PRISMA: Preferred Reporting Items for Systematic Reviews and Meta-Analyses.

^b^N/A: not applicable.

^c^QoL: quality of life.

^d^RCT: randomized controlled trial.

^e^iCBT: internet-based cognitive behavioral therapy.

^f^mHealth: mobile health.

^g^Item 23 (“Give results of additional analyses, if done [eg, sensitivity or subgroup analyses, meta-regression; see Item 16]”) was not applicable as no additional analyses were conducted in this review.

### Participant Characteristics

Reviews included data from an average of 1880 recruited participants (range 62-4084). One review included an analysis of online support groups representing 32,859 users in total. A total of 14 papers reviewed studies of cancer survivors of all disease sites, 3 papers included studies of breast cancer survivors, and 3 papers included studies of prostate cancer survivors. Table 1 provides further details on the population focus of each review.

A total of 7 reviews reported the gender breakdown of the included participants. Of these, 3 (43%) included only women, 2 (29%) included only men, and 2 (29%) were mixed gender (78% and 1034/1220, 84.8% women, respectively). Of the remaining 65% (13/20) reviews, inspection of the original included studies revealed that 6 (46%) included studies mostly or entirely comprised women. Descriptive data for participant demographic characteristics were reported in 13 reviews; the average age of participants in the original studies ranged from 26 years to 69 years, with the most common mean age being between 50 years and 59 years.

### Intervention Characteristics

The interventions reviewed included a range of self-guided, clinician-guided, and peer-led approaches, where some interventions combined 2 or more of these approaches into a multicomponent intervention. Most (33/40, 83%) interventions were hosted on self-guided web-based platforms, some of which also facilitated interactions with a clinician or peer group. Other intervention types included web-based videoconferencing with a clinician, online peer support groups, and mobile phone–based symptom management. The full list of interventions represented in the included reviews is available in Multimedia Appendix 3.

Most reviews included at least one study investigating CBT (15/20, 75% reviews), self-determination theory or self-management interventions (14/20, 70% reviews), or interventions focused on increasing access to social support (eg, online peer support groups; 11/20, 55% reviews). Other theoretical frameworks included mindfulness-based approaches, problem-solving therapy, self-efficacy theory, social exchange theory, self-regulatory therapy, therapeutic writing, representational approach, nontheoretically oriented web-based counseling, and combinations thereof (Multimedia Appendix 3). Where reviews focused only on original studies adopting a specific delivery format or theoretical framework, this has been specified in the “Intervention” column in Table 1. None of the reviews explicitly specified a theoretical backdrop in their approach to synthesizing original study data.

### Narrative Synthesis

#### Overview

A true thematic analysis was not possible, as there were insufficient narrative syntheses on uptake, engagement, and our efficacy outcomes of interest to facilitate the identification of convergent codes and themes. Rather than developing convergent codes, we instead categorized the findings and recommendations of each review directly under an inductive thematic structure. Following the initial generation of the thematic structure, themes and subthemes were subsequently audited independently by 2 separate authors to ensure integrity to the original qualitative data set. The final narrative structure was agreed upon through consensus. The following sections will discuss, in turn, the narrative themes identified for intervention uptake, intervention engagement, efficacy, factors promoting efficacy, and recommendations for future research.

#### Intervention Uptake

The facilitating factors and barriers to intervention uptake and engagement are summarized in Table 2. The dominant theme was difficulties with recruitment. Forbes et al [36] found that 67% (6/9) of the original studies that reported a recruitment goal did not meet their stated recruitment targets, resulting in underpowered analyses. Goliță and Băban [37] identified several person-related factors predicting greater likelihood of uptake, including education level, being female, being White, and breast cancer diagnosis. However, as 37% (7/19) of the original studies included in the review by Goliță and Băban [37] only included patients with breast cancer in their eligibility criteria, this finding should be interpreted with caution.

Barriers to intervention uptake were grouped under 2 themes: person factors and contextual factors. Person factors included *greater anxiety around technology*, and contextual factors included *perceived time burden* during an already-stressful period [36,42]. In addition, Moradian et al [42] highlighted that in an original study, 23% of the study participants never logged into the study intervention [51]. Follow-up interviews with the participants of this study revealed several explanations for nonuse of the intervention, including (1) perceived lack of need, given existing access to other resources; (2) preference for telephone or face-to-face over web-based communication with their health care provider; and (3) being put off by aspects of the intervention itself, such as log-in difficulties [59].

**Table 2:** Facilitating factors and barriers to intervention uptake and engagement^a^.

	Facilitating factors	Barriers
Uptake	Weak evidence for demographic factors Greater education Women White Breast cancer diagnosis	Anxiety around technology Perceived time burden
Engagement	Tailoring and customizability of the intervention Demographic factors Younger age Women Being married Greater experience with the internet Email messages and reminders	Difficulties with technology Participant clinical profile Greater fatalism Poorer coping with anxiety Less impairment caused by pain Perceived time burden Lack of satisfaction with the intervention

^a^Owing to the paucity of relevant quantitative data in the included reviews, the factors influencing uptake and engagement were identified by extracting narrative syntheses from each review.

#### Intervention Engagement

The reviews generally reported low dropout rates [35,44]. For example, Qan’ir and Song [44] reported retention rates between 73% and 94% in 8 studies, a retention rate of 31% in 1 study, and 1 study that did not report on participant dropout. Three major themes were found to facilitate intervention engagement: (1) *tailoring and customizability* of the intervention to meet specific needs, (2) participant *demographic* characteristics, and (3) *email messages and reminders*. Regarding tailoring to specific needs, Goliță and Băban [37] found that interventions focusing on a specific diagnosis or phase of the disease (eg, survivorship) had lower attrition. Greater ability of the participants to customize the intervention to meet their needs and more personalized feedback were also associated with greater retention [37,47]. These findings were supported by qualitative feedback, suggesting that greater provision of cancer-specific information and more personalized feedback, potentially supplemented by telephone or face-to-face contact, were preferred [45]. Regarding participant demographic characteristics, Paul et al [43] found that younger age, being female, being married, and previous experience with the internet predicted greater intervention use. Finally, Wang et al [47] suggested that email reminders may support greater engagement. The importance of including e-messages is further supported by evidence that e-messages and self-care advice are the components most commonly used by patients with low social support and high levels of symptom distress and depression [42,52].

Barriers to intervention engagement included (1) *difficulties with technology*, (2) *participant clinical profile*, (3) *time burden*, and (4) *lack of satisfaction* with the intervention. Paul et al [43] and Seiler et al [45] found that lower levels of computer literacy were associated with lower levels of intervention use. These issues were compounded in some studies by the requirement of additional software and a lack of clarity on how to use unfamiliar software [36]. Griffiths et al [17] highlighted a study, which found that greater fatalism, poorer coping with anxiety, and less impairment caused by pain were associated with a greater likelihood of participant dropout [53].

The finding that perceived time burden was a barrier to intervention use [45,47] maps closely onto similar findings discussed earlier with regard to intervention uptake and relates to reports by some participants that interventions were too difficult to integrate into their daily lives [45]. Finally, a lack of satisfaction with the intervention, including discrepancies with participant expectations [47], and a lack of perceived change in relationships and personal strengths [17] also predicted lower adherence.

#### Intervention Efficacy

##### Overview

The key efficacy findings for each study are reported in Table 1, and the summary efficacy findings for each included outcome variable are presented in Table 3. The findings are discussed in further depth in the following sections.

**Table 3:** Intervention efficacy: proportion of reviews reporting favorable results per outcome.

	Distress (n=9), n (%)	Depression (n=13), n (%)	Anxiety (n=5), n (%)	Stress (n=4), n (%)	Quality of life (n=17), n (%)	Well-being (n=4), n (%)
Mostly favorable outcomes^a^	3 (33)	10 (77)	1 (20)	4 (100)	6 (35)	1 (25)
Mixed findings^a^	1 (11)	1 (8)	3 (60)	0 (0)	2 (12)	0 (0)
Mostly null or negative findings^a^	5 (56)	2 (15)	1 (20)	0 (0)	9 (53)	3 (75)

^a^Mostly favorable outcomes are defined as a majority of studies finding at least significant pre-post effects. Mixed findings refer to reviews where 38% to 50% of the studies found significant pre-post effects. Mostly null or negative findings refer to reviews where <38% (3/8) of the included studies found positive effects.

##### Distress

In all, 33% (3/9) of the reviews found mostly favorable results for distress reduction, with most (5/9, 56%) reviews reporting mixed findings or null effects. Goliță and Băban [37] reported that, of the 6 out of 16 studies included in their review which reported clinically significant improvements in distress, only 17% (1/6) of the studies found maintenance of the improvements at the 6-month follow-up, 17% (1/6) of the studies found that benefits decreased over the 6-month follow-up, and 67% (4/6) studies did not investigate long-term maintenance of intervention effect. The results did not clearly indicate an optimum treatment stage or population for addressing distress using internet-based interventions [37]. Fridriksdottir et al [21] reported that of the 3 studies that found significant distress improvement in their review, all (3/3, 100%) were CBT-based interventions including automated information provision, monitoring, feedback, and self-management components.

##### Depression

A clear majority of reviews [17,34-36,42,43,45-48], including 3 meta-analyses [35,45,47], demonstrated positive effects on depression. Few (3/20, 15%) reviews included a narrative synthesis of efficacy results, with the exception that Forbes et al [36] highlighted a study, which found that web-based CBT was superior to an online chat forum [54]. In addition, Griffiths et al [17] highlighted that most studies reported moderate to large pre-post effect sizes for depression among women with breast cancer, although most of these studies did not include a control group.

##### Anxiety and Stress

One review reported mostly favorable effects of web-based psychosocial interventions for anxiety [46], 3 found mixed results [21,42], and 1 found mostly null results [44]. By contrast, all 4 reviews including stress as an outcome reported mostly favorable results. The narrative syntheses of the reviews did not offer an explanation for the superior effects found for stress versus anxiety. However, given that few original studies included anxiety and stress as outcome variables, future research is needed to clarify whether these findings indicate true differences in efficacy for these closely related constructs or whether reported efficacy differences may reflect confounding elements of study design.

##### Quality of Life

Reviews including a narrative synthesis of QoL improvements reported mixed evidence for efficacy. The review that included the largest number of relevant original studies found significant QoL improvement over a control group in 3 out of 10 (30%) studies that fell within the scope of this meta-review [37]. There were no clear intervention factors differentiating studies that found statistically significant effects from those that did not. Indeed, almost all studies investigated a CBT-based intervention, so comparison based on different theoretical frameworks was not possible. However, given that most studies found small to medium effect sizes favoring the web-based interventions, many of these studies may have simply been underpowered to detect small effect sizes.

##### Well-being

Evidence for the efficacy of web-based psychosocial interventions in improving well-being was weak, with only 1 out of 4 (25%) reviews that included well-being as an outcome finding mostly favorable evidence. The remaining reviews (3/4, 75%) mostly found null results. None of the reviews included a narrative synthesis that specifically pertained to well-being data.

### Factors Promoting Intervention Efficacy

We categorized the factors that moderate intervention efficacy into five themes: (1) *study outcomes*, (2) *intervention factors*, (3) *person factors*, (4) *study design*, and (5) *general uncertainty around significant moderating factors*.

#### Study Outcomes

Regarding study outcomes, 2 reviews reported better outcomes for pain [34] and distress [37] over QoL. Goliță and Băban [37] proposed that the weaker results reported for QoL may be a function of both the interventions and outcome measures used in the original studies. That is, almost all interventions under investigation were CBT-based, which Goliță and Băban [37] suggested may prioritize symptom management rather than broader QoL outcomes. Furthermore, inconsistencies in the measurement tools used, including some unvalidated measures for cancer survivors, may render them less appropriate for identifying clinically meaningful changes in this population.

#### Intervention Factors

There were conflicting findings regarding the efficacy of multicomponent versus single-component interventions. Specifically, Fridriksdottir et al [21] reported that multicomponent interventions were generally associated with superior outcomes for symptom distress, and Triberti et al [46] reported the same findings for QoL, emotional well-being, depression, stress, and anxiety. By contrast, Griffiths et al [17] reported that multicomponent interventions were associated with poorer outcomes for depression. However, given that the finding of Griffiths et al [17] arises from a meta-analysis including populations without a diagnosis of cancer, the findings of Fridriksdottir et al [21] and Triberti et al [46] may be considered more relevant to the aims of this meta-review.

Regarding specific intervention components, the data generally favor interventions that fostered greater communication with a health care professional. For example, the Comprehensive Health Enhancement Support System [55], in combination with remote support from an expert mentor, was generally effective in supporting a range of psychosocial outcomes [46]. Fridriksdottir et al [21] found that a nurse-facilitated email communication forum was the intervention component most valued by participants, being considered both more informative and easier to understand compared with other information provision components. These data suggest that efforts to improve the cost-effectiveness of interventions by minimizing clinician involvement must be balanced against the needs and wishes of cancer survivors.

Griffiths et al [17] assessed the potential moderating effects of several intervention factors that are specifically associated with web-based support. However, this meta-analysis largely yielded null results: intervention efficacy was not moderated by synchronous versus asynchronous chat room engagement, presence versus absence of a chat room moderator, public versus private nature of the support group, length of intervention duration, or length of follow-up. Indeed, the only factor moderating the intervention effect was the degree of engagement, such that greater levels of chat room posting were associated with improved mood. However, this finding is likely confounded by several person-related factors, including strength of motivation and positive expectations for the intervention effect, thus shedding little light on any inherent intervention features that better support psychosocial outcomes.

#### Person Factors

Several reviews have presented a narrative synthesis of person factors moderating intervention efficacy, including a review that conducted a meta-analysis across populations with and without cancer [17]. No clear demographic or sociodemographic factors emerged that were consistently associated with the intervention outcomes. An original study found that older age and greater baseline distress were associated with greater QoL improvements [60], whereas another original study found that younger age was associated with greater stress reduction [56]. Another original study found that higher emotional communication competence was associated with greater improvements in psychological QoL [57]. However, the overall evidence base assessing the suitability of web-based psychosocial interventions for subpopulations of cancer survivors is limited and is characterized by more null than positive findings.

#### Study Design

Griffiths et al [17] found a trend for low-quality studies to be associated with more positive outcomes among a clinically heterogeneous sample of people using internet support groups. However, this finding was not replicated in a more recent meta-analysis that focused exclusively on cancer survivors [45]. On balance, the current evidence therefore does not support a clear association between study quality and outcomes.

#### Uncertainty Around Moderating Factors

The dominant theme that emerged from the reviews was the lack of any identifiable factors that significantly moderated the intervention effect [37,40,41,44]. Several authors of included meta-analyses commented that there were too few directly comparable studies to enable subgroup analysis [40,47], whereas the meta-analyses that quantitatively investigated potential moderators largely failed to identify any statistically significant moderating person or intervention variables for the dependent variables under investigation in this meta-review [16,45]. Exceptions include the findings of Xu et al [16] that the type of control group and duration of the intervention significantly moderated the intervention effect. Comparison against a wait-list or usual-care control group was associated with more favorable effects than against other support controls. The direction of effect regarding study duration was not reported.

### Recommendations for Future Research

Our top recommendations for future research are summarized in Textbox 3, categorized according to five main themes: (1) study design, (2) reporting, (3) study outcomes, (4) study samples, and (5) interventions.

**Top recommendations for future research.**

**Study design**

- Conduct randomized controlled trials
- Conduct fully powered studies
- Investigate potential mediators of intervention effect
- Investigate potential moderators of intervention effect
- Include an active comparison group

**Reporting**

- Report study findings transparently, adhering to CONSORT (Consolidated Standards of Reporting Trials) guidelines
- Report rates of study uptake
- Report rates of participant engagement with the intervention and with data collection procedures

**Study outcomes**

- Use standardized, validated measures of common study outcomes
- Measure a broader range of outcomes, including patient empowerment, information support, and clinical outcome

**Study samples**

- Conduct studies across a broader range of national and cultural contexts
- Conduct further research among underserved communities
- Conduct further research in men with advanced cancer

**Interventions**

- Ensure that intervention content is guided by relevant theory
- Ensure ease of use across mobile and nonmobile devices
- Tailor interventions to specific populations or specific support needs

#### Study Design

The dominant recommendation to appear across most reviews was a need for a greater number of high-quality clinical trials [16,17,31,33-36,38,39,43-45,47,48]. There was a preponderance of pilot and feasibility studies, which were largely underpowered and often lacked a control group. The review authors thus highlighted a need for fully powered trials to move the evidence base beyond initial feasibility testing and toward efficacy testing of clinically significant benefits for patients and survivors [31,34,38,43,45].

#### Reporting

Several reviews have highlighted a need for more transparent reporting of clinical trials following the CONSORT (Consolidated Standards of Reporting Trials) guidelines [31,34], including more transparent and consistent reporting of participant intervention engagement [31,33,36,44,45].

The reviews highlighted a lack of investigation into potential mechanisms of intervention effects, with several authors recommending that mediation analyses should be factored into future study designs [21,37,38]. Moderation analyses, including dose-effect responses [21,37,44] and responses to different intervention modalities [33,38,44] are also required. Reviews have also recommended head-to-head clinical trials comparing different types of web-based psychosocial interventions [33,37,43] to more conclusively determine which form of interventions work best and for whom. This would add to the robustness of trial conclusions, given that wait-list control participants are often less likely to seek contemporaneous support, given the anticipation of future therapeutic benefit from the intervention under investigation [30,37]. Paul et al [43] emphasized the importance of ensuring that head-to-head trials are adequately powered to enable subgroup analyses; for example, to assess differential intervention effects for participants with lower versus higher levels of socioeconomic advantage.

#### Study Outcomes

Reviews also highlighted the need to use validated and standardized measures of common study outcomes (eg, distress, depression, and QoL) so that different clinical trials can be directly compared [34,41]. Reviews have also recommended expanding the scope of future studies to investigate a broader range of outcomes, such as fatigue, empowerment, information support, knowledge, biomarkers of clinical distress (eg, proinflammatory cytokines and salivary cortisol), long-term clinical outcomes, and patient satisfaction [38,44,45,48].

#### Study Samples

Reviews commonly recommended that future studies should recruit more heterogeneous populations of cancer survivors [37,38,44], particularly across different national and cultural contexts [38,45,48]. Hong et al [38] specifically noted a lack of research conducted within historically underserved communities, recommending special attention be paid to assessing literacy needs and ensuring the cultural appropriateness of interventions targeting low socioeconomic and minority cultural groups. Finally, reviews also highlighted a literature gap related to men with advanced cancer [36] and suggested that future studies should investigate the moderating effect of gender [44] and disease site [40] on intervention efficacy.

#### Interventions

The final category of research recommendations relates to the characteristics of the web-based psychosocial interventions under investigation. Recommendations within this category fall into three subcategories: (1) *theoretical considerations*, (2) *intervention modalities*, and (3) *tailoring of interventions to specific needs*. Regarding theoretical considerations, the authors highlighted the importance of ensuring that the intervention content was guided by relevant psychological theory [21,48]. Furthermore, McAlpine et al [41] highlighted the importance of developing a framework for the *process* of developing interventions following a rational approach to compiling intervention content based on recent evidence and the specific needs of the targeted population. Several reviews have recommended that future studies should investigate the utility of app-based psychosocial interventions [31,45] and ensuring ease of use across both mobile and nonmobile devices [45]. It is important to ensure that all intervention platforms are sufficiently user-friendly [44] and able to evolve in line with developments in technology and updates in relevant research [48]. Finally, reviews commonly recommended ensuring that interventions are tailored to the needs of specific patient and survivor groups [33,37], including different phases of cancer treatment and recovery [38,47]. In line with study design recommendations related to investigating mechanisms of effect, reviews also suggested that future studies seek to identify which components of study interventions are necessary to support specific psychosocial and supportive need outcomes [21,33,42,44,47].

## Discussion

### Summary of Findings

#### Overview

This meta-review aimed to identify and critically appraise the existing systematic reviews of web-based psychological and psychosocial interventions for adult patients with cancer and cancer survivors. Specifically, our objectives were to identify the factors that support the uptake, engagement, and efficacy of web-based psychosocial interventions for patients with cancer and cancer survivors. A lack of consistency and transparency in reporting uptake and engagement data in the original intervention studies stymied the ability of previous systematic reviews to identify a consistent set of facilitating factors and barriers to intervention uptake and engagement. Nonetheless, we identified some preliminary themes from the few reviews that reported a narrative synthesis of patterns in participant uptake and engagement.

#### Factors Associated With Recruitment

Many original studies reported difficulties with study recruitment, leading to analyses that were ultimately underpowered [36,37]. Only one review offered a summary of person factors associated with a greater likelihood of intervention uptake, which included greater levels of education, being female, being White, and a breast cancer diagnosis [37]. However, the fact that studies targeting breast cancer were overrepresented in this review poses an important confounder to the interpretation of these data, and we would therefore caution against firm conclusions regarding the predictive power of these demographic factors for intervention uptake.

Two key barriers to recruitment, however, did clearly emerge from the narrative data: (1) individual anxiety about technology and (2) perceived time burden of the intervention [36,42]. Promisingly, these factors can feasibly be addressed by study teams seeking to support recruitment in future web-based psychosocial oncology interventions. For example, authors have previously suggested the possibility of allowing participants to *reduce* or *expand* content to suit their preferences for the amount and depth of content they would like to engage with [61]. Although this suggestion was originally made with the aim of meeting participants’ information monitoring needs, advertising this capability may also address participants’ concerns about the time burden of web-based psychosocial interventions.

The authors have also highlighted the importance of using a simple and intuitive interface to support participant interest and engagement [62]. In line with these recommendations, we recommend that interventions are co-designed in an iterative manner with research partners with lived experience who are demographically representative of the target population [61]. This component of intervention design is important to optimize interventions before significant resources are invested in conducting randomized clinical trials investigating these interventions.

#### Factors Associated With Engagement

In contrast to poor levels of intervention recruitment, studies have generally reported high levels of participant retention [35,44]. Overall, participants engage more with interventions tailored to a specific need set and which allow a greater degree of personalization. Thus, although generic interventions aimed at a heterogeneous range of chronic illnesses appear to carry the benefits of general relevance and subsequent cost savings, any such benefits must be weighed against participant preferences for specificity and likelihood of use. Qualitative data highlighted participant preferences for intervention customizability, personalized feedback, and e-messages, which offer a potential solution to common perceptions concerning the impersonal nature of web-based interventions. Nevertheless, greater clinician involvement carries a clear additional resource cost and must be weighed against demonstrable clinical benefits. Offering participants the opportunity to customize the intervention to meet their needs is a technical feature that can be readily built into most intervention platforms and thus represents a simple, cost-effective way to increase the likelihood of intervention engagement.

Regarding clinical profile, Griffiths et al [17] highlighted a study that found that greater fatalism, poorer coping with anxiety, and less impairment caused by pain were associated with a greater likelihood of participant dropout [53]. It is unsurprising that the participants with greater levels of fatalism would hold less hope for the utility of continuing to engage with the study intervention. In addition, one can speculate that participants with lower coping abilities may have required more intensive one-on-one therapy to see benefits rather than the low-level electronic group support offered in the study intervention [52]. At first glance, it may appear counterintuitive that lower levels of impairment caused by pain were associated with higher levels of dropout. One possible explanation is that this finding reflects a lower level of need for support with physical and psychological concerns associated with pain management. Nevertheless, these findings should be treated with caution given that they were endorsed by only one original study.

Furthermore, 50% (2/4) of the top-cited barriers to engagement, difficulties with technology and time burden, are notably shared in common with our list of barriers to intervention uptake. Thus, the strategies highlighted earlier to address these barriers bear additional importance to successfully maintain intervention engagement after initial study consent. Wang et al [47] also highlighted that a lack of satisfaction with specific interventions was associated with a greater likelihood of dropout, emphasizing the importance of qualitative research to better understand the needs, expectations, and preferences of target cancer survivor groups. Researchers can subsequently use this information to minimize discrepancies between participant expectations and actual features of these interventions.

#### Factors Associated With Intervention Efficacy

Evidence for the efficacy of web-based psychosocial interventions for patients with cancer and cancer survivors was highly mixed, with significant variation between the different patient-reported outcomes included in this review. Overall, reviews have consistently endorsed web-based interventions for reducing depressive symptoms. Indeed, all meta-analyses including depression as an outcome variable found significant improvements compared with controls [35,45,47]. Reviews have also endorsed mostly favorable findings for addressing stress symptoms, although the evidence is relatively weaker given that fewer original studies have investigated stress. By contrast, the evidence for distress, anxiety, QoL, and well-being is weak to mixed, at best. However, this finding should be interpreted in the context of the paucity of studies investigating anxiety and well-being to date and in light of the inconsistency of the measures chosen to assess distress. Therefore, in future research, it would be useful to measure all 4 outcomes using a consistent battery of psychometric tests. For example, the 3 most commonly used measures of psychological distress in patients with cancer are the Profile of Mood States-Short Form [63], Distress Thermometer [64], and Hospital Anxiety and Depression Scale [65], all of which would yield results that are directly comparable with a large number of previous studies [66].

By contrast, QoL was the most consistently reported outcome across reviews, with 35% (6/17) of the reviews reporting favorable outcomes, 12% (2/17) of the reviews reporting mixed results, and 53% (9/17) of the reviews reporting null or negative results. Only one meta-analysis found a statistically significant benefit for health-related QoL [45], whereas the remaining 3 meta-analyses investigating overall QoL reported no significant differences between the intervention and control groups [16,35,47]. Overall, the existing evidence synthesized in this meta-review does not support the efficacy of web-based psychosocial interventions in supporting general QoL among cancer survivors. However, given the noted inconsistency in the methods used to measure QoL across the original intervention studies, with some studies using measures not explicitly created for populations living with and beyond cancer [37], it is premature to conclude that QoL is not affected by web-based interventions. Rather, future research needs to ensure that QoL is assessed using consistent, validated measures to ensure the validity of the research findings. In addition, it may be useful to explore facets of QoL separately to better identify the benefits that web-based psychosocial interventions may hold for specific domains of functioning [45].

Few conclusive factors associated with superior intervention efficacy were identified. With the exception of intervention duration [16], none of the meta-analyses identified any intervention features that significantly moderated the intervention effect [16,35,45,47]. However, our narrative synthesis provides preliminary indications that (1) multicomponent interventions and (2) interventions facilitating internet-based clinician contact are associated with superior outcomes. To ensure the most efficient use of health care resources, it would be useful to incorporate health economic analyses into future clinical trials to determine whether low-level web-based clinician support in combination with other internet-based content (eg, a self-guided website or online support group) may produce more cost-effective benefits than traditional face-to-face support. Further, RCTs investigating head-to-head comparisons of different web-based psychosocial interventions, or different variations of web-based psychosocial interventions, remain necessary to yield conclusive evidence regarding which features of web-based programs work best and for whom.

### Recommendations for Future Research

Our top 5 recommendations for future research are, first, for a greater number of fully powered RCTs, to enable more robust conclusions about the efficacy of web-based psychosocial oncology interventions. Second, we recommend that authors of future studies report study uptake, engagement, and study outcomes transparently, adhering to CONSORT guidelines. Third, we recommend the use of outcome measures that have been validated within the target population, with a preference for measures commonly used in previous research to support a more coherent and robust evidence base. Fourth, we recommend investigating web-based psychosocial intervention effects in a broader range of patient populations, including understudied national and cultural cohorts and men. Finally, we recommend interventions that are directly targeted at specific diagnostic groups or support needs, including customizable feedback and features, to encourage greater intervention engagement.

### Strengths and Limitations

This meta-review had several strengths, including our ability to identify and account for inconsistencies in the recommendations of previous relevant systematic reviews, resulting in a comprehensive overview of the efficacy of web-based psychosocial interventions for populations living with and beyond cancer. Synthesizing the recommendations of previous reviews has facilitated the compilation of a clear and commonly endorsed set of research recommendations to advance the field of eHealth in psychosocial oncology. Nevertheless, very few reviews have synthesized data on participant uptake and engagement with web-based interventions, rendering our recommendations in these domains tentative, pending further evidence.

With regard to the limitations of this review, our narrative approach to synthesizing previous review findings has limited our ability to conclusively comment on the statistical significance of variables reported to be associated with the uptake, engagement, and efficacy of web-based psychosocial oncology interventions. Nevertheless, we aimed to transparently report the findings of previous quantitative meta-analyses where present, while also comprehensively reporting on the full range of review findings to date, including where these findings are not commensurable with quantitative aggregation.

### Conclusions

Our meta-review supports the efficacy of web-based psychosocial oncology interventions for depression and stress, but there is currently insufficient evidence for distress, anxiety, QoL, and well-being. Future research can seek to promote both intervention uptake and engagement by addressing participant anxiety about technology and perceived time burden. Existing evidence suggests that multicomponent interventions and web-based clinician contact promote intervention efficacy. Future studies including head-to-head comparisons, which are fully powered to conduct subgroup analyses, are needed to conclusively establish what works best for maximizing recruitment, engagement, and efficacy.

## Appendix

Multimedia Appendix 1 Search strategy and data extraction form.

Multimedia Appendix 2 PRISMA (Preferred Reporting Items for Systematic Reviews and Meta-Analyses) checklist for included studies.

Multimedia Appendix 3 Breakdown of original studies and intervention types.

## Abbreviations

CBT: cognitive behavioral therapy
CONSORT: Consolidated Standards of Reporting Trials
PRISMA: Preferred Reporting Items for Systematic Reviews and Meta-Analyses
QoL: quality of life
RCT: randomized controlled trial
