# Supplementary material for: Web-Based Psychological Interventions for People Living With and Beyond Cancer: Meta-Review of What Works and What Does Not for Maximizing Recruitment, Engagement, and Efficacy
Source: JMIR Cancer. 2022 Jul 8;8(3):e36255. doi: 10.2196/36255 (PMC9308073; doi:10.2196/36255)
Supplement: Multimedia Appendix 1 [file cancer_v8i3e36255_app1.docx]

**Supplementary Material**

*Search Strategy*

We searched for the following terms in PubMed: ((Neoplasms[MeSH Terms]) AND ((telemedicine[MeSH Terms]) OR (Internet-Based Intervention[MeSH Terms])) AND (review[Title/Abstract])). An overview of what MeSH terms are and topics included within our main subject terms is presented below. We applied an English language filter in the PubMed search. Based on slight differences in the tree structure of subject headings used in CINAHL, we searched for Neoplasms (exploded) and Telehealth (exploded) in a subject heading search, combined with the word “review” in the abstract. Based on the limited number of reviews pertaining to telehealth or telemedicine in the Cochrane library, we searched for all reviews pertaining to the MeSH Term “Telemedicine (exploded)”.

In PsycINFO, we searched for the following terms as a Keyword or within the article Title or Abstract: ((oncol* OR neoplasm OR cancer OR tumour OR tumor OR leuk* OR lymphoma OR myeloma) AND (review OR meta-anal* OR “research synthesis”) AND ((digital OR internet* OR web OR online OR computer* OR technolog* OR app OR mobile OR telecommunication) AND (intervention OR treatment OR therap* OR psycho* OR “mental health” OR cognitive OR behavior* OR behaviour* OR mindful* OR emotion* OR distress* OR “quality of life")) OR (telehealth OR telemedicine OR eHealth OR mHealth OR uHealth)). We also applied an English language filter to the PsycINFO database search. We subsequently screened the reference lists of articles identified as eligible for inclusion to identify other eligible articles.

*PubMed MeSH Terms*

Medical Subject Headings (MeSH) terms constitute a hierarchical vocabulary system used by the National Library of Medicine to index journal articles contained within the MEDLINE and PubMed databases. The purpose of MeSH terms is to provide a consistent way to label and organise journal articles pertaining to similar concepts, despite potential differences in the terminology used within individual articles. MeSH Terms are allocated by subject specialists and continually updated. Please see the following link for the full tree structure associated with the exploded MeSH term “Neoplasms”: <https://meshb.nlm.nih.gov/record/ui?ui=D009369>. The following link contains details of the full tree structure associated with the exploded MeSH term “Telemedicine”: <https://meshb.nlm.nih.gov/record/ui?ui=D017216>. Finally, the following link contains details of the full tree structure associated with the exploded MeSH term “Internet-Based Interventions”: <https://meshb.nlm.nih.gov/record/ui?ui=D000079382>.

*CINAHL Subject Headings*

Similar to MeSH Terms, CINAHL subject headings constitute the hierarchical vocabulary used within the CINAHL database. Each item within the database is associated with a set of subject terms used to describe the content of that article. The “Neoplasms” subject heading used in our search includes topics related to cysts, neoplasms of every site, neoplasms of every histologic type, neoplastic processes and syndromes, and precancerous conditions. The “Telehealth” subject heading used in our search includes topics related to telemedicine, telenursing, and telepsychiatry. Full tree structures of our chosen CINAHL subject headings can viewed via a CINAHL Subject Heading search within the CINAHL database.

**Data Extraction Form**

| **General Information** | Author |  |
| --- | --- | --- |
|  | Year Published |  |
|  | Title of Study |  |
| **Methods** | Type of review (Systematic review with narrative synthesis or meta-analysis?) |  |
|  | Systematic review question |  |
|  | Year Systematic Search Was Conducted |  |
|  | Time Range of Systematic Search |  |
|  | Population focus |  |
|  | Interventions reviewed |  |
|  | Outcomes captured |  |
|  | Full inclusion criteria |  |
|  | Full exclusion criteria |  |
|  | **Complete PRISMA Checklist Below ** | Number of Items Present:  Number of Items Not Present:  Number of Items Not Applicable: |
| **Findings** | Total number of participants included in review |  |
|  | Gender Breakdown of entire participant sample |  |
|  | Descriptive age data for entire participant sample |  |
|  | Level of bias reported across studies |  |
|  | Uptake of interventions reviewed | Range:  Mean rate of uptake:  Median rate of uptake:  SD rate of uptake:  Narrative description of levels of uptake: |
|  | Narrative reporting of facilitating factors and barriers to recruitment and uptake |  |
|  | Adherence to interventions reviewed | Range:  Mean rate of adherence:  Median rate of adherence:  SD rate of adherence:  Narrative description of levels of adherence: |
|  | Narrative reporting of facilitating factors and barriers to intervention adherence |  |
|  | Efficacy of interventions reviewed as compared to control conditions | Range:  Mean efficacy effect size:  Median efficacy effect size:  SD rate of efficacy effect size:  Narrative description of intervention efficacy: |
|  | Narrative reporting of factors that promote intervention efficacy and suitability of outcome measures |  |
| **Conclusions** | Recommendations for future research |  |

**Data Extraction Form (Continued)**

**PRISMA Checklist: Indicate whether each item is present in review, and the page number on which the information can be found**

| **Section/topic** | **#** | **Checklist item** | **Present? (Y/N)** | **Reported on page #** |
| --- | --- | --- | --- | --- |
| **TITLE** | | |  |  |
| Title | 1 | Identify the report as a systematic review, meta-analysis, or both. |  |  |
| **ABSTRACT** | | |  |  |
| Structured summary | 2 | Provide a structured summary including, as applicable: background; objectives; data sources; study eligibility criteria, participants, and interventions; study appraisal and synthesis methods; results; limitations; conclusions and implications of key findings; systematic review registration number. |  |  |
| **INTRODUCTION** | | |  |  |
| Rationale | 3 | Describe the rationale for the review in the context of what is already known. |  |  |
| Objectives | 4 | Provide an explicit statement of questions being addressed with reference to participants, interventions, comparisons, outcomes, and study design (PICOS). |  |  |
| **METHODS** | | |  |  |
| Protocol and registration | 5 | Indicate if a review protocol exists, if and where it can be accessed (e.g., Web address), and, if available, provide registration information including registration number. |  |  |
| Eligibility criteria | 6 | Specify study characteristics (e.g., PICOS, length of follow-up) and report characteristics (e.g., years considered, language, publication status) used as criteria for eligibility, giving rationale. |  |  |
| Information sources | 7 | Describe all information sources (e.g., databases with dates of coverage, contact with study authors to identify additional studies) in the search and date last searched. |  |  |
| Search | 8 | Present full electronic search strategy for at least one database, including any limits used, such that it could be repeated. |  |  |
| Study selection | 9 | State the process for selecting studies (i.e., screening, eligibility, included in systematic review, and, if applicable, included in the meta-analysis). |  |  |
| Data collection process | 10 | Describe method of data extraction from reports (e.g., piloted forms, independently, in duplicate) and any processes for obtaining and confirming data from investigators. |  |  |
| Data items | 11 | List and define all variables for which data were sought (e.g., PICOS, funding sources) and any assumptions and simplifications made. |  |  |
| Risk of bias in individual studies | 12 | Describe methods used for assessing risk of bias of individual studies (including specification of whether this was done at the study or outcome level), and how this information is to be used in any data synthesis. |  |  |
| Summary measures | 13 | State the principal summary measures (e.g., risk ratio, difference in means). |  |  |
| Synthesis of results | 14 | Describe the methods of handling data and combining results of studies, if done, including measures of consistency (e.g., I^2^) for each meta-analysis. |  |  |
| **Section/topic** | **#** | **Checklist item** | **Present? (Y/N)** | **Reported on page #** |
| Risk of bias across studies | 15 | Specify any assessment of risk of bias that may affect the cumulative evidence (e.g., publication bias, selective reporting within studies). |  |  |
| Additional analyses | 16 | Describe methods of additional analyses (e.g., sensitivity or subgroup analyses, meta-regression), if done, indicating which were pre-specified. |  |  |
| **RESULTS** | | |  |  |
| Study selection | 17 | Give numbers of studies screened, assessed for eligibility, and included in the review, with reasons for exclusions at each stage, ideally with a flow diagram. |  |  |
| Study characteristics | 18 | For each study, present characteristics for which data were extracted (e.g., study size, PICOS, follow-up period) and provide the citations. |  |  |
| Risk of bias within studies | 19 | Present data on risk of bias of each study and, if available, any outcome level assessment (see item 12). |  |  |
| Results of individual studies | 20 | For all outcomes considered (benefits or harms), present, for each study: (a) simple summary data for each intervention group (b) effect estimates and confidence intervals, ideally with a forest plot. |  |  |
| Synthesis of results | 21 | Present results of each meta-analysis done, including confidence intervals and measures of consistency. |  |  |
| Risk of bias across studies | 22 | Present results of any assessment of risk of bias across studies (see Item 15). |  |  |
| Additional analysis | 23 | Give results of additional analyses, if done (e.g., sensitivity or subgroup analyses, meta-regression [see Item 16]). |  |  |
| **DISCUSSION** | | |  |  |
| Summary of evidence | 24 | Summarize the main findings including the strength of evidence for each main outcome; consider their relevance to key groups (e.g., healthcare providers, users, and policy makers). |  |  |
| Limitations | 25 | Discuss limitations at study and outcome level (e.g., risk of bias), and at review-level (e.g., incomplete retrieval of identified research, reporting bias). |  |  |
| Conclusions | 26 | Provide a general interpretation of the results in the context of other evidence, and implications for future research. |  |  |
| **FUNDING** | | |  |  |
| Funding | 27 | Describe sources of funding for the systematic review and other support (e.g., supply of data); role of funders for the systematic review. |  |  |

*From:*  Moher D, Liberati A, Tetzlaff J, Altman DG, The PRISMA Group (2009). Preferred Reporting Items for Systematic Reviews and Meta-Analyses: The PRISMA Statement. PLoS Med 6(7): e1000097. doi:10.1371/journal.pmed1000097

For more information, visit: **www.prisma-statement.org**.
